# Acceptability of a Positive Parenting Programme on a Mother and Baby Unit: Q-Methodology with Staff

**DOI:** 10.1007/s10826-016-0564-9

**Published:** 2016-10-06

**Authors:** Hannah Butler-Coyne, Dougal Hare, Samantha Walker, Angelika Wieck, Anja Wittkowski

**Affiliations:** 1 School of Health Sciences, Division of Psychology and Mental Health, University of Manchester, 2nd Floor Zochonis Building, Brunswick Street, Manchester, M13 9PL UK; 2 Psychology, Cardiff University, Cardiff Wales, UK; 3 Department of Psychiatry, Manchester Mental Health and Social Care Trust, Manchester, England, UK; 4 Department of Clinical Psychology and Mother and Baby Unit (Andersen Ward), Manchester Mental Health and Social Care Trust, Manchester, England, UK

**Keywords:** Positive parenting programme, Baby Triple P, Mother and baby unit, Mothers with severe mental illness, Q-methodology

## Abstract

The Baby Triple P Positive Parenting Programme, a new addition to the established Triple P programmes, is currently being considered for a trial in a Mother and Baby Unit with the aim of exploring its benefits to mothers presenting with severe mental illness. The aim of the current study was to investigate staff views of the acceptability and feasibility of a parenting programme such as the Baby Triple P Positive Parenting Programme in a Mother and Baby Unit. Q-methodology, using an 88-item Q-sort, was employed to explore the opinions of 16 staff working in a Mother and Baby Unit in the North West of England. Results obtained from the Q-sort analysis identified two distinct factors: (1) *staff qualified acceptance* and (2) *systemic approach/systemic results*. Preliminary findings indicate that staff perceived Baby Triple P to be an acceptable and feasible intervention for the Mother and Baby Unit setting and that mothers on the unit would be open and receptive to the programme. Further research is required to expand these findings and assess the potential for this type of intervention to be used more widely across a number of Mother and Baby Unit settings.

## Introduction

Research has explored the potential effects of parents experiencing serious mental health issues on their child’s development (O’Donnell et al. 2014; Breaux et al. 2013; Sanders 2012; Goodman et al. 2011; Fraser et al. 2006) with adverse effects reported for the quality of the attachment relationship (Martins and Gaffan 2000) and the child’s socio-emotional and cognitive development (Feldman et al. 2009). Mothers with severe mental illness (SMI) face the difficult and additional challenge of negotiating their own mental health difficulties alongside adjusting to their (new) role of being a mother (Dolman et al. 2013; National Institute for Clinical Excellence [NICE] 2007). Although mothers with SMI place particular emphasis on the role of being a mother (Dolman et al. 2013), studies have shown that certain parenting practices are compromised, specifically for mothers with postnatal depression, including the mother–infant interaction (Arteche et al. 2011), maternal responsiveness (Milgrom et al. 2004) and attentiveness (Minkovitz et al. 2005). Parenting approaches adopted by families with SMI vary widely and the limited practice- and research-based evidence within this area offers little guidance. However, there is clearly an urgent need for effective and targeted parenting interventions for this specific population (O’Hara 2009; Dolman et al. 2013). This is in contrast to the emerging data on the effectiveness of parenting interventions in alleviating such problems in other contexts (see Sanders et al. 2014).

As some mothers with SMI, including schizophrenia and severe depression, require specialist psychiatric services (Oyserman et al. 1994), within the United Kingdom admission to Mother and Baby Units (MBUs) may be warranted. These units are designed to meet the mental health treatment needs of the mother and to provide specialist therapeutic services and access to multi-disciplinary healthcare staff (NICE 2007), advice and support with childcare, including the consistency and adequacy of parenting skills (Seneviratne et al. 2003). Admissions can be voluntary or involuntary under the Mental Health Act; they can occur antenatally but most commonly occur postnatally. Often the mother is admitted having spent some time with her baby at home unless onset of her mental health difficulties occurred immediately after birth (as in psychosis after childbirth).

Despite various studies highlighting the potential impact of parental mental health difficulties on child development (O’Connor et al. 2014; Beardslee et al. 2011; Goodman et al. 2011), there appears to be a lack of evidence on the effectiveness of parenting interventions for mothers with severe mental illness in general and especially within a MBU setting. This lack of evidence may be due to interventions focussing on treating the mental illness first and foremost, which is entirely appropriate during an inpatient admission. Consequently, as yet there is no clinical guidance relating to parenting interventions in MBUs (NICE 2007), although parenting skills training is regarded as “desirable” (Royal College of Psychiatrists 2008).

The Triple P (2013) Positive Parenting Programmes, based on social and developmental theories, have been developed as preventively oriented parenting and family support strategies (Sanders et al. 2003, 2012, 2014). This evidence-based multi-level intervention system ranges from low intensity (Level 1), a universal parenting programme directed towards increasing general knowledge of parenting information, to high intensity (Level 5), an enhanced and specialised programme for parents experiencing significant stressors (e.g., mental health problems and high levels of stress) (Sanders et al. 2003). It is based on five core principles (*safe and engaging environment, positive learning environment, assertive discipline, realistic expectations* and *parental self-care*) and all levels can be accessed depending on individual needs and circumstances (Thomas and Zimmer-Gembeck 2007; Sanders 2012).

Research evidence is emerging exploring the benefits of different formats of Triple P in a mental health context. For example, in their pilot study of a web-based application of Triple P for parents with bipolar disorder, Jones et al. (2013) reported apparent improvements in child behaviour and perceived parenting but the authors qualify their findings due to the study characteristics (e.g., pilot study, sample size, etc.). Another pilot randomised controlled trial evaluated the benefits of the Baby Triple P Positive Parenting Programme (Baby TP) for mothers with postnatal depression (Tsivos et al. 2014) against treatment as usual. Whilst the mothers receiving Baby TP showed more favourable improvements, both groups improved so that no significant differences were noted. However, Baby TP was rated as highly acceptable to mothers with PND.

Baby TP (Level 4) is a new form of Triple P; it aims at developing the knowledge, skills and confidence of parents. The need for parenting skills training resulted in one MBU in North West England evaluating the usefulness of Baby TP on an exploratory basis.

Staff perspectives as to the feasibility and acceptability of psychological or psychiatric interventions allow for the exploration of views on the delivery of care and multi-faceted challenges, which can lead to the resolutions of issues pertinent to the population groups (Dolman et al. 2013; NICE 2007). Whilst there is some research into the acceptability of Triple P (Kirby and Sanders 2013) as well as Baby TP (Tsivos et al. 2014; Butler et al. 2014), there is a paucity of research into staff views about working with mothers presenting with SMI and on the effectiveness and accessibility of parenting interventions in these settings (David et al. 2011, Dolman et al. 2013). Acceptable and feasible interventions involve accessibility, applicability, sustainability and achievability (Breitenstein 2013; Dahlgren et al. 2013; Stallard and Buck 2013); hence, using Q-methodology, the aim of the current study was to examine the acceptability and feasibility of a parenting programme, such as Baby TP, to staff working on a MBU.

## Method

### Participants

The sample comprised of staff working within a 10-bedded, specialist, multi-disciplinary (psychiatrists, mental health nurses, nursery nurses, support workers and clinical psychologist), inpatient Mother and Baby Unit (MBU) in the North West of England for mothers presenting with moderate to severe mental health issues (such as psychosis and severe affective disorders), who are admitted jointly with their infant, provided the child is below the age of 12 months old.

A convenience participant sample (P-Set) comprised of 16 female MBU staff members: five nursery nurses (31 %), four staff nurses (25 %), one senior staff nurse (6 %), one support worker (6 %), one ward manager (6 %), one assistant ward manager (6 %), two specialist trainee psychiatrists (13 %) and one consultant psychiatrist (6 %). Length of experience ranged from two months to 34 years (M = 14.8 years; SD = 10.9). All staff approached agreed to take part. Participants did not offer any manualised or evidence-based parenting or psychological interventions to patients during the study period.

Most staff (n = 13) stated that their knowledge regarding parenting for this specific population came from ‘general knowledge and ward experience’, with three reporting knowledge being informed by studying for a psychology degree, knowledge of Webster-Stratton parenting techniques and the Parent–Child–Game and attending a brief, informal, in-house presentation to staff informing them about the Triple P principles and the format of the Baby TP programme. The breadth, depth and quality of participant opinions are more relevant than sample size in Q-methodology (Brown 1996), and the general rule of having fewer participants than Q-set items was followed (Watts and Stenner 2012).

### Procedure

The study received approval from the National Health Service Research Committee (REC Reference 11/NW/0716) and the National Health Service Research and Development department (R&D Reference 1091).

#### Design

In the absence of established questionnaires to obtain staff views regarding parenting interventions specific to MBU settings, Q-methodology (Stephenson 1953) was identified as an appropriate approach to obtain subjective opinions of participants in exploratory research, when little is known about a particular issue (Watts and Stenner 2005). Specific advantages of Q-methodology include being able to recruit a larger, but still manageable, sample of staff within a shorter time than would be possible using qualitative techniques (Smith 2001). Furthermore, Q-methodology permits identification of multiple subjective viewpoints without the risk of undue researcher bias. Comparable studies of acceptability and feasibility of new treatments and interventions have demonstrated the appropriateness of using Q-methodology in this manner (e.g., Butler et al. 2014).

#### Q Methodology Phase 1—Concourse and Q-Set Development

As per standard Q-set development, relevant literature was reviewed, including studies of views, attitudes and opinions on acceptability and feasibility of parenting programmes, together with extant satisfaction questionnaires, evaluation proformas and Q-sets concepts. Additionally, semi-structured interviews were conducted with one MBU service user and two members of staff. Members of the Quality Network for Perinatal Mental Health and the Division of Clinical Psychology Faculty of Perinatal Psychology were contacted via e-mail and posting on Network and Division forums inviting professionals with expertise in the field to provide views and opinions about the acceptability and feasibility of parenting interventions. These all contributed to the development of a Q concourse reflecting a full and balanced range of views and opinions within this area (Watts and Stenner 2012).

The first author (HB-C) and a clinical psychologist (SW) identified 636 independent items, which were grouped into 68 similar themes, resulting in an 88-item Q-set. Items were allocated a random number and printed onto laminated cards for subsequent Q-sorting by participants. To check comprehension, pilot Q-sorts were conducted with two clinical psychologists and a former service user. The method and statements were deemed suitable with no further changes required.

#### Q Methodology Phase 2—Administering Q-Sets and Obtaining the Q-Sorts

Sixteen MBU staff members, provided with information about the Baby TP research, irrespective of their knowledge about the programme, completed the 88-item Q-set by reading through the Q-statements and initially sorted them into piles: disagree, neutral and agree. Then they systematically ranked statements, according to a forced choice distribution, by how strongly they rated the statement on an opinion continuum (Q-grid) (+6 strongly agree to −6 strongly disagree). Post-sort interview questions were asked about Q-statements rated at the extreme ends of the Q-grid, as well as about the Q-process. The Q-sort interview is a critical, yet often overlooked, part of the Q-methodology process (Brown 1980). Interviewing participants provides valuable information about their wider understanding of how and why they have rated items in a particular way, whilst enabling them to expand on the personal meaning and significance of their rating choices (Watts and Stenner 2012). Overall the Q-sort task took between 45–60 minutes to complete.

### Data Analyses

Q-sort analysis was conducted using PQMethod (2.11) (Schomolck and Atkinson 2002). Factor extraction was conducted using principle component analysis (PCA) with a varimax rotation to maximise variance within the factors. Q-sort relationships were initially presented in a correlation matrix. Seven (unrotated) factors were extracted using PCA, with two factors (accounting for the largest amount of variance) subjected to varimax rotation. The resultant analysis identified defining Q-sorts, factor scores, factor arrays (exemplar sorts) and consensus statements (Watts and Stenner 2012).

## Results

Ten Q-sorts loaded onto Factor 1 (26 % variance) and six loaded onto Factor 2 (20 % variance) (Table 1). Factor 1 was the “strongest” factor because it accounted for the largest rotated variance.

**Table 1:** Factor arrays for the two factors

No	Statement	Factor 1	Factor 2
1	The MBU provides time to take part in Baby TP	1	2
2	It is important that Baby TP fits nicely with the ethos of the unit	0	−1
3	The skills taught in Baby TP need to generalise to environments other than the MBU	3	6
4	When mothers are unwell, Baby TP will be intolerable	−1	−3
5	Mental health issues prevent mothers from accessing Baby TP	−3	−4
6	Baby TP needs to fit with the mothers’ mental health	4	−1
7	Baby TP should be flexible to the mothers mental health status	2	3
8	Baby TP will be fluid and flexible	−1	0
9	Baby TP will be flexible to cope with unplanned events	0	4
10*	The facilitator needs to be skilled in their explanation of Baby TP	1	1
11	Staff rolling out Baby TP need to have a thorough knowledge about mother and baby	1	−2
12	Baby TP will make women’s anxieties about their ability to parent worse	−3	−4
13	It is important for staff to be able to answer questions about Baby TP	4	2
14	Baby TP is a reactive response from “anxious” professionals	−5	−4
15	Staff need to believe that Baby TP benefits the mother	1	0
16	The techniques of Baby TP flow through to the staff on the MBU	−2	1
17	It is important that all staff know which mothers are using the Baby TP techniques	2	−1
18	It is important that the mother thinks Baby TP is worthwhile	4	1
19	It is important that mothers are open to change	2	4
20	If the mother has unchangeable situations at home, Baby TP is not going to be helpful	−4	−6
21	Mothers want to be recognised for the work they are doing in Baby TP	0	2
22	Baby TP is “preachy”	−5	−3
23	People providing Baby TP should only suggest techniques	−1	−3
24	A trusting relationship with the Baby TP therapist is important	5	3
25	One-to-one work will make it easier for mothers to say when they find Baby TP difficult	5	1
26	If the relationship between the Baby TP facilitator and mother is not working, neither will Baby TP	−1	0
27*	It is important that Baby TP complements what staff already know	−1	−1
28*	Doing Baby TP will make mothers feel like “bad parents”	−6	−6
29	Baby TP might make people feel like they are being unfairly judged or blamed	−4	−3
30	It is important that mothers doing Baby TP can gauge their progress	4	2
31*	It is important for mothers to feel they have achieved something	5	5
32	Baby TP is an extra thing to engage in and will make mothers feel overwhelmed	−4	−5
33	It is OK for Baby TP to be challenging for mothers	0	3
34	Mothers should have ongoing support in doing Baby TP	3	2
35	It is important that the mother’s family are open to change	−1	0
36	Baby TP will help develop skills that can help deal with family problems	−1	6
37*	It is important that Baby TP sessions do not interfere with family visits on the MBU	−2	−2
38	It is important that mothers feel in control and responsible for Baby TP	2	0
39	Well delivered Baby TP will maintain overall confidence in the MBU	1	0
40	Baby TP will be helpful for mothers to meet their parenting needs	2	6
41	If a mother is severely depressed, they will not have the motivation to do Baby TP	1	−2
42	Whilst staying on the MBU it is easy for mothers to commit to Baby TP	−2	2
43*	In order to engage in Baby TP, staff expect mothers to be open to learning	0	0
44	Baby TP will use all the mothers energy and focus	−6	−4
45	It is important that the Baby TP therapist works with both mother and baby	4	−3
46	Baby TP will address mothers feelings of uncertainty	0	1
47	It is important that Baby TP engages with the current situation and needs of the mother	6	4
48	It is important that mothers have a positive attitude towards recovery from illness	2	1
49*	Taking part in Baby TP will be a positive experience	1	1
50*	It is important that mothers discuss Baby TP with other like-minded people	−3	−3
51*	There is no opportunity to practice the Baby TP skills on the MBU	−6	−6
52*	It is important that Baby TP is easy for mothers to do	−1	−1
53*	Baby TP will be about what has gone wrong for mother and baby	−5	−5
54	Baby TP needs to emphasise the positive so as not to make the mother’s mental illness worse	2	3
55	The way Baby TP is presented to mothers will be important	3	0
56	It is important that Baby TP does not go against what mothers already know	−1	−4
57*	It is important for the mother to recognise what she has done well	5	5
58*	It is important for the mother to recognise what she could have done differently	2	2
59*	It is important for the mother to recognise what she has done wrong	−2	−2
60	Staff need to think about what parts of the Baby TP would be helpful for mothers	3	0
61	It is important to encourage staff to reflect	0	3
62	Staff need support and training to feel confident in delivering the Baby TP skills	6	3
63	All staff should have the same training in Baby TP	1	0
64*	It is important that both staff and mothers will find Baby TP enjoyable	1	1
65	Staff have too much work to do to support Baby TP skills adequately	−4	−5
66	Baby TP will be easily incorporated into the workload of staff	−2	−1
67	Baby TP should not have too much paperwork for staff to do	−3	2
68*	It is important that Baby TP only takes a small amount of staff time	−3	−3
69	Baby TP should be a priority for the MBU	−2	2
70	Baby TP should not get in the way of other MBU work	−3	−2
71	Baby TP is about learning new skills	0	1
72	Baby TP provides a safe place for mothers who have mental health issues	−2	−1
73	It is important Baby TP will highlight the importance of mothers looking after themselves	0	5
74	Engagement with mothers must be the priority in Baby TP	3	5
75	Doing Baby TP would make mother’s feel exposed or a bad mother	−4	−5
76	Staff attitude affects engagement on Baby TP	1	4
77	Mothers want factual information about parenting	0	4
78	Practical materials are essential	−2	3
79	Baby TP comes at the wrong time	−5	0
80	The Baby TP therapist needs to really sell the programme to mothers	−3	−1
81	It is important for all staff on the MBU to have a clear role within Baby TP	3	−2
82*	All staff should support what is done in Baby TP	−1	−1
83	It is important that mothers and Baby TP therapists work together to solve the mother’s problems	2	1
84	It is important that staff understand why Baby TP works	6	0
85	Mothers being able to make choices in Baby TP is important	3	−1
86	Mothers should decide when they want to do Baby TP sessions	0	−2
87	Mothers need to know what they can do and cannot do for Baby TP to work	0	−2
88*	Baby TP needs to be based on common-sense	−2	−2

Consensus statement [statements 10; 27; 28; 31; 37; 43; 49; 50; 51; 52; 53; 57; 58; 59; 64; 68; 82; 88]

An exemplar or idealised Q-sort for each of the two factors was developed using a factor array (Table 2) (Watts and Stenner 2012) to provide detailed information for factor descriptions, aided by the participants’ post Q-sort interview.

**Table 2:** Participant demographics and factor loading

Factor loading	No.	Professional title	Experience (Years)	Previous knowledge of parenting interventions
1	2	Nursery nurse	17	General knowledge and ward experience
1	3	Staff nurse	14	Degree in Psychology
1	4	Nursery nurse	31	General knowledge and ward experience
1	5	Assistant ward manager	2.5	General knowledge and ward experience
1	6	Staff nurse	3	General knowledge and ward experience
1	9	Nursery nurse	23	General knowledge and ward experience
1	10	Senior staff nurse	32	General knowledge and ward experience
1	11	Nursery nurse	9	General Knowledge and ward experience
1	12	Nursery nurse	34	Baby TP presentation and general knowledge
1	14	Staff nurse	0.16	General knowledge and ward experience
2	1	Psychiatry—specialist trainee	7	General knowledge and ward experience
2	7	Ward manager	7	General knowledge and ward experience
2	8	Support worker	10	General knowledge and ward experience
2	13	Staff nurse	5.5	General knowledge and ward experience
2	15	Psychiatry—specialist trainee	6	General knowledge and ward experience
2	16	Consultant psychiatrist	22	Webster Stratton, parent-child game

### Factor 1: Staff Qualified Acceptance

This factor accounted for 26 % of the variance and represented the majority of the P-set (n = 10, 63 %), encompassing a diverse range of experience (from two months to 34 years), professional and general parenting experience (Table 1) and was labelled *‘Staff Qualified Acceptance’* to reflect staff positive beliefs about implementing a parenting programme like Baby TP on the MBU and potential staff training needs. Staff who loaded onto this factor regarded this type of intervention as an accessible parenting approach for mothers with SMI and disagreed with potential detrimental effects of the intervention by endorsing the following statements: “Baby TP will use all the mother’s energy and focus” (−6; 44), “Baby TP will make women’s anxieties about their ability to parent worse” (−3; 12) and “Baby TP is an extra thing to engage in and will make mothers feel overwhelmed” (−4; 32). However, staff indicated that delivery of a parenting programme should focus on the circumstances of the mother and her current mental health: “It is important that Baby TP engages with the current situation” (+6; 47). Staff agreed strongly that “one-to-one work will make it easier for mothers to say when they find Baby TP difficult” (+5; 25), whilst emphasising that “a trusting relationship with the Baby TP therapist is important” (+5; 24).

Staff loading onto this factor reported a lack of knowledge regarding the content and concepts of Baby TP with strong agreement that “it is important that staff understand why Baby TP works” (+6; 84) and that “staff need support and training to feel confident in delivering the Baby TP skills” (+6; 62). (NB only the MBU clinical psychologist [AWit] was trained in Baby TP at this stage of its implementation). This was reflected in the neutral response to statements requiring opinions on the content of the intervention, such as “Baby TP is about learning new skills” (71). Staff marginally disagreed with “the techniques of Baby TP flow through to the staff on the MBU” (−2; 16), but they agreed with “it is important that all staff know which mothers are using the Baby TP skills” (+2; 17). Staff commented further about their support for a parenting intervention and expressed their wish to become more aware of the programme:

“I don’t know as much as I would like to know about Baby TP but my understanding is that it is driven from a needs-led perspective, from mums’ perspective and it is not about us, it is about helping people to be successful parents.” (Participant 3)

“…We all need to be singing from the same song sheet although I think it should be done by one main person really, who is trained.” (Participant 11)

### Factor 2: Systemic Approach/Systemic Results

This factor accounted for 20 % of the variance and represented six participants (37 %) ranging in experience (5.5–22 years), profession and parenting knowledge (Table 2). All staff with psychiatric training (n = 3) loaded onto this factor. The label of ‘*Systematic Approach/Systematic Results*’ reflected staff beliefs that what the service users learned from a parenting programme should address and enhance all elements of a mother’s life and not just parenting skills per se.

Staff loading onto this factor supported the implementation of a parenting programme, reporting strong beliefs about the benefits and positive outcomes for the mother with strong agreement for “Baby TP will be helpful for mothers to meet their parenting needs” (+6; 40). Staff also strongly agreed with the statement that “it is important Baby TP will highlight the importance of the mother looking after herself” (+5; 73). They also strongly agreed that “the skills taught in Baby TP need to generalise to environments other than the MBU” (+6; 3) and “Baby TP will help develop skills that can help deal with family problems” (+6; 36). Equally, staff strongly believed that the mother’s circumstance did not negatively affect the benefits of the programme: “if the mother has unchangeable situations at home, Baby TP is not going to be helpful” (−6; 20) and disagreed that “mental health issues prevent mothers from accessing Baby TP” (−4; 5).

Comments made by staff offer additional support for the implementation of a parenting programme and re-iterated the systemic effects of such an intervention:

“I think it is like with any talking or psychological therapy, if you can only do it in the session or in a particular situation and you can not translate it into your everyday life, it would not be very useful.” (Participant 16)

“I think that you would be hoping that they would be able to take these skills and use them in the community so it is not just about coping whilst they are in hospital but also with life in general.” (Participant 15)

### Consensus Statements

In addition to the two factors, the Q-analysis identified 17 consensus statements equally rated across the factors (Brown 1996). All staff believed that a parenting programme would be a positive intervention for mothers with SMI with strong disagreement that “doing Baby TP will make mothers feel like ‘bad parents’“ (−6; 28) and “Baby TP will be about what has gone wrong for mother and baby” (−5; 53). Staff strongly endorsed the statements that “it is important for mothers to feel they have achieved something” (+5; 31) and “it is important for the mother to recognise what she has done well” (+5; 57). They also suggested that the focus of a parenting programme should be on the positive aspects of the mother’s parenting, mildly disagreeing with “it is important for the mother to recognise what she has done wrong” (−2; 59), whilst equally agreeing with “it is important for the mother to recognise what she could have done differently” (+2; 58). Staff believed that the MBU setting was ideal for a parenting programme by rejecting the statement “there is no opportunity to practice the Baby TP skills on the MBU” (−6; 51). They mildly disagreed with the statement that “it is important that Baby TP complements what staff already know” (−1; 27). Staff acknowledged the importance of a parenting programme on the MBU, disagreeing that “it is important that Baby TP only takes a small amount of staff time” (−3; 68).

### Comparison between Factors 1 and 2

The two factors were significantly correlated (r = 0.67), indicating a strong positive relationship and agreement regarding the acceptability and feasibility of a parenting programme like Baby TP.

## Discussion

Q-sorts with 16 MBU staff members indicated that they regarded a parenting programme like Baby TP to be a feasible and acceptable intervention that would be favourably perceived by service users. Indeed, services users admitted to the same unit viewed this parenting programme as such (Butler et al. 2014). MBU staff indicated that such a parenting programme did not reflect negative characterisation as a response to the “bad parent” stigma often identified in the population of mothers presenting with SMI (Dolman et al. 2013). According to staff responses, achievements gained through involvement in a parenting programme enhanced mothers’ self-esteem, which was viewed positively because maternal self-esteem is often low due to maternal feelings of failure and inadequacy associated with their situation (NICE 2007; David et al. 2011). In terms of feasibility of implementation, staff viewed the MBU ward environment as being conducive and able to accommodate a parenting programme.

The main factor emerging from the Q-sort data has been termed ‘*Staff Qualified Acceptance*’ due to their positive attitudes towards the benefits of a parenting programme like Baby TP. Staff believed that one important aspect of the intervention was that the facilitator spent individual time with mothers working on parenting skills. The need for a flexible delivery was also recognised as being important to accommodate for changing (mental health and situational) circumstances. This does align with Triple P principles and the aim to provide a flexible and individually tailored intervention. The importance in establishing a therapeutic relationship as a means to empower families and build upon resiliencies is recognised as essential in Triple P programmes (Sanders et al. 2003).

If staff on the unit are to offer this approach more widely, they must feel confident in parenting skills and techniques to ensure that the programme is able to be supported beyond the therapy room (Shapiro et al. 2012). Given that successful intervention implementation is closely linked to communication and collaboration between staff and agencies (Dolman et al. 2013), it is important that this is addressed prior to implementation of any intervention.

The second factor, labelled ‘*Systemic Approach/Systemic Results*’, reflected the view of staff that any parenting intervention should address all areas of a mother’s life with the skills being transferable to different life situations. This also aligns with the ethos of Triple P programmes that focus on promoting the wellbeing of the family, enhancing the competence, resourcefulness and self-sufficiency of parents and reducing the incidence of maternal difficulties with severe mental health issues (Triple P, www.triplep.net). Views of staff with psychiatric training backgrounds were particularly prominent within this factor, which reflects the holistic approach of their clinical training. How differential views of staff with particular training influence results is worthy of further investigation.

### Methodological Limitations

Whilst the current findings are encouraging, there are methodological issues. Although large sample sizes are not necessary in conducting a Q-analysis (McKeown and Thomas 1998), the contextually bound, relatively small sample size of 16 MBU staff members limits generalisation of the findings and further research is needed to extend and broaden the sample base. Participant biases, as a result of a perceived need to provide socially desirable responses, alongside some knowledge of Baby TP can influence outcome. Measures taken to reduce the likelihood of this included anonymity of responses, multiple opportunities throughout the Q-process for participants to clarify any issues or ask questions, and post-sort interview to discuss the Q-statements to address concerns.

### Clinical Implications

Given the lack of existing evidence, national guidance for parenting interventions within MBUs (NICE 2007; RCP 2008) is limited through non-endorsement of any particular parenting approach for mothers with SMI. In the current study, three main clinical implications were identified, which may contribute to the development of an evidence base of appropriate interventions for this population. Firstly, a parenting programme like Baby TP was viewed positively by staff and, from a staff perspective, the programme would be feasible and acceptable within this clinical setting. Indeed, mothers with postnatal depression (Tsivos et al. 2014) and with SMI (Butler et al. 2014) viewed Baby TP as highly acceptable. Secondly, Baby TP was provided as a therapist-facilitated individual intervention by the MBU clinical psychologist (AWit) ﻿and﻿ staff recognised their own lack of knowledge about the programme and their desire to have known more about it in order to advise their service users about it. It would be advantageous for service users if staff were more knowledgeable about the guiding principles behind the parenting programme on offer so that staff could support service users by translating and generalising the skills and techniques used within the sessions across the MBU. It was interesting to note that half of the participants loading onto Factor 2 had psychiatric training. Thus, the form and extent of staff training requires careful consideration, particularly in an environment where the demands on resources and staff time are at a premium. Staff case discussion groups and designated time slots within clinical meetings to focus on parenting information may be a way of cascading the principles into the MBU environment, together with efforts to promote measures to review the compliance and success of adoption by staff (Turner et al. 2011). Thirdly, the current findings support previous recommendations for more specialist education and training of staff working in MBUs (Dolman et al. 2013), in particular for working with mental health difficulties and stigma on which staff have received minimal guidance (Bower and Gilbody 2005).

### Future Research

Exploring larger and more diverse samples, longer term assessment of the outcomes, time and cost-effectiveness of implementation of a parenting programme in MBU settings, as perceived by both staff and service users, would provide additional information regarding feasibility and acceptability, as well as validating the approach and informing clinical practice. The acceptability and feasibility of a parenting programme like Baby TP on a MBU from the service user perspective has already been examined and offers insight into the service user-specific opinions within this same setting (Butler et al. 2014). In conclusion, a parenting programme like Baby TP appears to be an acceptable, feasible and valued intervention for use within a specialist mental health context.
